# Barriers to Physical Activity During Pregnancy and Postpartum—A Narrative Review

**DOI:** 10.3390/healthcare14060793

**Published:** 2026-03-20

**Authors:** Józef Opara, Jarosław Szczygieł, Katarzyna Szczygieł

**Affiliations:** 1 Institute of Physiotherapy and Health Sciences, Jerzy Kukuczka Academy of Physical Education, Ul. Mikołowska 72b, 40-065 Katowice, Poland; j.szczygiel@awf.katowice.pl; 2 Department of Neurology, Clinical Hospital No. 1 Named After Prof. Stanisław Szyszko, Ul. 3 Maja 13/15, 41-800 Zabrze, Poland; 3 Institute of Psychology, Humanitas University, Ul. Kilińskiego 43, 41-200 Sosnowiec, Poland; 4 Department of Neurology, Upper Silesian Medical Center Named After Prof. Leszek Giec, Ul. Ziołowa 45/47, 40-635 Katowice, Poland

**Keywords:** physical activity, barriers, pregnancy, postpartum period, lifestyle, exercise, determinants, review

## Abstract

This article addresses physical activity during pregnancy and the postpartum period, a crucial public health concern. We examine the latest insights into physical activity during the perinatal phase, highlighting key findings on the attitudes, perceived barriers, and factors that influence participation. Engaging in moderate physical activity during this period is deemed safe and offers numerous benefits, such as lowered risks of gestational diabetes, preeclampsia, and excessive weight gain, alongside enhanced mental health and sleep quality. After childbirth, continued physical activity provides advantages such as weight management, reduced postpartum depression risk, improved sleep patterns, and a better overall quality of life. However, activity during these stages is often hindered by various barriers stemming from personal issues, societal influences, knowledge gaps, and environmental obstacles. Notably, these challenges tend to shift between pregnancy and postpartum; safety concerns are more prevalent during pregnancy, while issues like fatigue, lack of time, and childcare responsibilities become more significant after delivery. This article uses a socio-ecological framework to analyze these obstacles in depth, categorizing them into intrapersonal, interpersonal, environmental, organizational, and policy-based levels.

## 1. Introduction

Physical activity during pregnancy and the postpartum period is undeniably an important public health concern. In 2019, DiPietro et al., representing the Physical Activity Guidelines Advisory Committee, shared the findings of a systematic review analyzing the benefits of physical activity during these stages [1]. Drawing on 76 systematic reviews and meta-analyses conducted between 2006 and 2018, the study highlighted compelling evidence that engaging in moderate-intensity physical activity helps lower the risk of excessive gestational weight gain, gestational diabetes, and postpartum depressive symptoms. There was also limited evidence pointing to potential links between physical activity and reduced risks of preeclampsia, gestational hypertension, antenatal anxiety, and depressive symptoms. However, it remains unclear whether factors such as age, race, socioeconomic status, or pre-pregnancy weight play a mediating role in these associations, with most researchers addressing physical activity during pregnancy in women aged 16 to 49 years. Additionally, the term “perinatal period” has been introduced to encompass the timeframe spanning the 12 months before conception, pregnancy itself, and the postpartum period, usually defined as the year following childbirth [1,2].

We recently published an article on recommendations for lifestyle physical activity and exercise during the perinatal period, concentrating on lifestyle PA [2].

## 2. Material and Methods

This narrative review was conducted to identify all relevant English-language randomized controlled trials (RCTs) available in PubMed, the Cochrane Library, Embase, and Web of Science published between 1983 and 28 February 2026 concerning physical activity (PA), particularly leisure-time physical activity (LTPA) and lifestyle physical activity (LPA). For this purpose, medical subject headings (MeSH) and full-text searches were employed, involving terms related to pregnancy, postpartum period (12 months after delivery), barriers, and determinants. Although the postpartum period lasts from six to eight weeks, in this case we took into account the view of Zielke et al. that regular physical activity is important for health maintenance across the lifespan and is particularly important during the perinatal period (1 year before conception to 1 year after childbirth) [2,3].

The exclusion criteria included the following: (1) studies involving participants facing issues unrelated to PA barriers in pregnancy and the postpartum period; (2) studies with incomplete datasets or lacking statistical analysis; and (3) studies without control groups or only including single protocols, abstracts, or conference poster presentations. While structured exercises and physical therapy were considered secondary areas of interest, the primary focus was PA’s role in PD prevention.

In total, 156 articles were initially identified through the search process, of which 18 met the inclusion criteria for this review.

## 3. Recommendations for Physical Activity During Pregnancy and Postpartum

In their 2026 review article, Opara et al. offered comprehensive recommendations regarding physical activity during pregnancy, highlighting its benefits, contraindications, and necessary precautions [2]. They made a distinction between general physical activity and structured exercise, referencing Dunn et al.’s 1998 definition of “lifestyle physical activity” (LSPA)—engaging in at least 30 min of often spontaneous activities, encompassing recreational, occupational, or household efforts performed at moderate to vigorous intensity as part of daily routines [2,4]. Studies by Torbé et al. and Nascimento et al. have indicated that women’s awareness of the importance of and possibilities for physical exercise during pregnancy remains insufficient [5,6]. Supporting this insight, the National Health and Nutrition Examination Survey (NHANES) conducted by Hesketh and Evenson revealed that 60% of American women surveyed did not participate in any form of leisure-time physical activity (LTPA) [7]. In 2009, Fell et al. showed that most pregnant women tend to experience reduced physical activity levels, with this decline during early pregnancy influenced by factors such as being under 35 years old, having multiple children, lower education levels, excess weight, and limited activity before conception [8]. Tailoring exercise regimens to each trimester is advised, with extra vigilance recommended during the first trimester [8], with recreational practices like stationary cycling, swimming, yoga, Pilates, running, and Nordic walking specifically noted for their positive impact on overall health during pregnancy [2].

Official guidelines on physical activity during pregnancy have been issued in nine countries: Australia, Denmark, France, Spain, Japan, Canada, Norway, the United Kingdom, and the United States. Most of these guidelines advocate for moderate-intensity physical activity throughout pregnancy, offering specific recommendations on the frequency and duration of exercise. Additionally, six of the guidelines outline both absolute and relative contraindications to physical activity. Across the board, activities associated with heightened fall risk, injuries, or collisions are generally discouraged, with six of the guidelines further specifying conditions under which exercise during pregnancy should be discontinued [9].

The American College of Obstetricians and Gynecologists (ACOG) recommended engaging in 20–30 min of exercise on most days of the week, ideally on a daily basis [10]. Five years later, they released an updated statement incorporating the latest research on the benefits and potential risks of physical activity during pregnancy and postpartum [11]. In 2023, a study by Gascoigne et al. revealed that nearly 48% of pregnant women in the United States exceed the recommended weight gain during pregnancy [12].

Most recommendations advise against engaging in sports and recreational activities under certain conditions during pregnancy, such as high-risk or multiple pregnancy, pregnancy-induced hypertension, bleeding, anemia, gestational preeclampsia, pyelonephritis, polyhydramnios, or oligohydramnios, while pregnant individuals with diabetes should approach exercise with extreme caution. Other contraindications to physical activity include poor general health, heart disease, fetal complications, a history of premature birth, and separation of the pubic symphysis. The Taking Test (TT) and the Borg Rating of Perceived Exertion (RPE) scale, which measures exercise intensity based on subjective feelings of effort, breathlessness, and fatigue, are recommended as methods for subjective assessment of physical exercise intensity [13,14]. Movements like deep squats and toe-touching bends are discouraged, as are exercises performed in a supine position, particularly after the fifth month of pregnancy. Lifting weights is not recommended during the third trimester, and exercise should cease immediately if symptoms like lower abdominal pain, headaches, or shortness of breath occur. Additionally, from the 20th week onward, exercises targeting the rectus abdominis muscles should be avoided; focus should instead be on strengthening the transverse and oblique muscles [2]. According to the Canadian guidelines written by Mottola et al. (2019), physical activity in extreme heat or high humidity should be avoided, diving is not recommended, and women residing at altitudes below 2500 m should refrain from physical activity at higher altitudes [15]. Table 1 and Table 2 present absolute and relative contraindications to exercise throughout pregnancy [15]. A Cochrane systematic review by Muktabhant et al. (2015) concluded that a balanced approach to diet and exercise can effectively prevent excessive weight gain during pregnancy [16].

The 2020 guidelines on physical activity and sedentary behavior issued by the World Health Organization (WHO) highlight the benefits of physical activity for pregnant and postpartum women. Regular exercise during these periods positively impacts both maternal and fetal health, helping to reduce the risks of conditions such as preeclampsia, gestational hypertension, gestational diabetes, excessive weight gain during pregnancy, postpartum depression, and peri- and neonatal complications. Furthermore, engaging in physical activity is not linked to negative effects on birth weight or an increased risk of stillbirth, with guidelines strongly recommending that all pregnant and postpartum women without contraindications incorporate regular physical activity into their routines. To achieve meaningful health benefits, it is advised to engage in at least 150 min of moderate-intensity aerobic exercise per week, as supported by moderate-certainty evidence, while for overweight or obese pregnant women, high-certainty evidence suggests that physical activity significantly reduces gestational weight gain and the risk of gestational diabetes. Additionally, further moderate-certainty evidence indicates a slight but meaningful reduction in the likelihood of preterm birth among mothers who participate in vigorous exercise. After giving birth, exercise should be reintroduced gradually, and women who have undergone a cesarean section are advised to consult a healthcare provider before resuming physical activity [17].

In their presentation of the Australian guidelines, Brown et al. (2022) concluded that a review of the scientific evidence confirms that perinatal PA and exercise are safe, offer health benefits to both the mother and unborn child, and may help reduce the risk of certain pregnancy complications [18]. Four specific recommendations highlight the need for exercise modifications to accommodate the physical changes experienced during pregnancy, as well as the importance of performing pelvic floor muscle exercises during and after pregnancy. Additionally, the guidelines identify warning signs and contraindications related to exercise and physical activity during pregnancy [18].

Key findings from the systematic review by Budlers et al. (2022) include a summary of 30 guidelines published in 11 languages [19], supported by Hayman et al. (2023) [20], while Evenson et al. (2024) [21] provided a review of global public health guidelines addressing physical activity and sedentary behavior during the postpartum period, specifically six to 12 weeks after childbirth.

## 4. Barriers to Physical Activity for Women During Pregnancy and the Postpartum

The question remains as to why, despite increasing awareness of the benefits of physical activity, many pregnant women tend to reduce their levels of physical activity. One possible explanation lies in identifying the barriers that hinder physical activity during pregnancy and the postpartum period. These obstacles can be categorized as objective or subjective, as well as individual-dependent or -independent, with particular emphasis on environmental and ideological factors. For instance, barriers may include medical contraindications such as bleeding, hypertension, or placenta praevia; warning signs like abdominal pain, shortness of breath, or amniotic fluid leakage; or issues like fatigue, pain, lack of time, and concerns regarding the safety of both mother and baby.

The review of studies examining barriers to physical activity during the perinatal period is organized chronologically, with foundational reports beginning in 2009. In that year, Evenson et al. asked a total of 1535 pregnant women in North Carolina (USA) between 27 and 30 weeks of gestation an open-ended question about their primary barrier to physical activity, with responses categorized based on the socio-ecological model [22]. Among the respondents, 85% identified intrapersonal barriers, with nearly two-thirds of these being related to health issues. Conversely, only 2% reported interpersonal barriers as their main challenge, while 3% highlighted neighborhood or environmental barriers [22].

Additionally, Cramp and Bray (2009) conducted a prospective study focused on exercise and barrier self-efficacy in relation to leisure-time physical activity (LTPA) during pregnancy [23]. This research involved 160 pregnant women who completed surveys addressing LTPA barriers, physical activity levels, barrier self-efficacy, and a six-week recall of LTPA at 18, 24, 30, and 36 weeks of gestation. Through content analysis of 1168 identified barriers, nine primary themes emerged, including fatigue, time constraints, and physical limitations. The study found that exercise self-efficacy was predictive of LTPA between both 18 and 24 weeks, and 30 and 36 weeks of gestation, whereas barrier self-efficacy predicted LTPA from 24 to 30 weeks [23].

In their systematic review, Coll et al. (2017) identified 12 quantitative and 14 qualitative studies spanning 1986 to 2016 [24]. The most commonly reported barriers were categorized as intrapersonal under the socio-ecological model, further divided into five key themes: (1) pregnancy-related symptoms and limitations; (2) time constraints; (3) Perceptions toward being active; (4) lack of motivation; and (5) concerns regarding maternal and child safety. At the interpersonal level, two primary themes emerged: (1) insufficient advice and information and (2) limited social support. Additionally, two overarching themes were identified at the environmental, organizational, and political levels: (1) adverse weather conditions and (2) inadequate resources. The accompanying table details these three main levels of barriers to leisure-time physical activity during pregnancy as outlined by the socio-ecological model [24].

Table 3 presents main levels of socio-ecological model barriers impacting leisure-time physical activity among pregnant women [23,24,25,26,27,28,29,30,31,32,33,34].

Analyzing the proportions of quantitative and qualitative studies according to the type of barriers reported, Coll et al. found that the most noticeable barriers to LTPA among pregnant women reported in the quantitative data were pregnancy-related symptoms and limitations and time constraints (100%), whereas among the qualitative data, the most frequently reported barriers were pregnancy-related symptoms, and limitations and lack of social support (100%). As for pregnancy-related symptoms and limitations the authors mentioned fatigue, tiredness, lack of energy, felling unwell or, uncomfortable, nausea, back and pelvic pain, swelling, soreness, shortness of breath, leg cramps, morning sickness, contractions, headache, anemia, disease, bodily changes, the growing body, and physical limitations. As for lack of motivation they named lower self-efficacy or discipline, pregnancy being a time to rest, a dislike of exercise, no habit of exercise, no pre-pregnancy physical activity routine, problems with body-image, embarrassment about appearance. Among lack of advice and information (interpersonal) they mentioned lack of knowledge about how to exercise safely during pregnancy, lack of healthcare provider guidance or counseling, lack access to consistent information, advice and support on the benefits of physical activity during pregnancy, insufficient and contradictory information, lack of accessible information [24].

Zulfiqar et al. (2017) investigated the obstacles that impeded the adoption of long-term healthy lifestyle practices among women diagnosed with gestational diabetes mellitus (GDM) in a study group of 18 Australian-born and 53 overseas-born women [25]. Both groups encountered numerous challenges in improving their post-GDM lifestyle, and although the participants retained information about healthy lifestyle recommendations, many struggled to recall those intended for long-term maintenance. Key barriers identified included the timing of health information delivery, inadequate reinforcement of lifestyle recommendations, and the uncoordinated and fragmented support offered by postpartum healthcare systems. For overseas-born women specifically, additional impediments were evident, such as the absence of culturally competent health education materials, cultural preferences regarding diet and exercise, and insufficient support from family members and spouses. Nevertheless, both Australian- and overseas-born participants demonstrated good adherence to their initial annual postpartum oral glucose tolerance test, attributed to their personal motivation coupled with reminders provided by healthcare professionals. Notably, many women resumed a healthier lifestyle only after childbirth, primarily aiming for weight reduction [25].

Harrison et al.’s (2018) systematic review incorporated 49 articles presenting data from 47 studies, encompassing a total of 7655 participants, with data collected from questionnaires, interviews, and focus groups [26]. The meta-analysis’ findings indicated that pregnant women generally held favorable attitudes towards physical activity, viewing it as essential, advantageous, and safe. However, the primary barriers to engaging in physical activity were intrapersonal in nature, including issues such as fatigue, time constraints, and pregnancy-related discomfort. Facilitating factors highlighted in the review included the perceived health benefits for both mother and fetus (intrapersonal), social support systems (interpersonal), and the availability of programs tailored specifically for pregnant individuals. Notably, only a limited number of environmental factors were identified [26].

Similarly, Makama et al. (2021) undertook a systematic review aimed at exploring the barriers and facilitators influencing the adoption of a healthy lifestyle during the first two years postpartum, as perceived by women and healthcare professionals [27]. Their analysis encompassed 28 studies employing both qualitative and quantitative methodologies, and categorized the identified barriers and facilitators into three key domains: capacity, opportunities, and motivation. Capacity-related factors included a lack of knowledge regarding the benefits of healthy lifestyle behaviors and limitations in healthcare professionals’ abilities to provide adequate support. Opportunities were shaped by factors such childcare demands and social support from partners, family members, friends, and healthcare providers. Lastly, motivational factors involved recognizing the benefits of physical activity and healthy eating, deriving enjoyment from such behaviors, and perceiving improvements in health. Their findings underscore the need for postpartum lifestyle interventions that effectively address these identified themes to support women’s health during this critical period [27].

Edie et al. (2021) conducted a systematic review of qualitative and quantitative studies to explore barriers to PA among postpartum individuals, with ten studies—seven qualitative and three quantitative—meeting their inclusion criteria and the identified barriers grouped into five categories: intrapersonal, interpersonal, sociocultural/demographic, physical environment, and healthcare environment [28]. The most commonly reported obstacles within these categories included fatigue and/or lack of sleep (7 out of 10 studies), limited time or unpredictable routines/schedules, heavy household and caregiving responsibilities (both noted in 8 out of 10 studies), insufficient support from family, friends, and other mothers (9 out of 10), weather conditions (7 out of 10), and breastfeeding (3 out of 10). The researchers emphasized the need for further studies incorporating more diverse postpartum populations that examine the impact of policy on postpartum physical activity levels [28].

Rhodes et al.’s (2021) online and telephone interview study explored the role of partners in influencing energy balance behaviors among pregnant women, further aiming to identify barriers to and facilitators of couples’ engagement in a digital intervention promoting healthy eating and physical activity during pregnancy [29]. The study population consisted of women who were either in the final trimester of pregnancy or had at least one child under 18 months of age, with most participants belonging to socioeconomically disadvantaged groups, where maternal obesity is more prevalent. The identified barriers and facilitators were analyzed using the COM-B (Capability, Opportunity, Motivation—Behavior) and Theoretical Domains Frameworks, with four primary themes emerging: partner involvement and support, partner understanding of healthy energy balance behaviors, couple adherence to energy balance behaviors, and the influence of partners on these behaviors. Notably, the majority of facilitating factors fell under the “Reflective Motivation” domain of the COM-B model. Male participants were primarily driven by a desire to act as supportive partners and positive role models, while female participants were motivated by the belief that their partners’ engagement would enhance their likelihood of achieving health goals and strengthen their relationship dynamics. Additional facilitators included shared eating habits (Physical Opportunity), recommendations from healthcare professionals, perceiving pregnancy as a shared experience (Social Opportunity), and feelings of mutual support and partnership (Automatic Motivation). While barriers were infrequently reported, they included potential conflicts with partners, perceiving pregnancy as solely the woman’s responsibility, and economic limitations. These findings underscore the complex interplay of motivational and contextual factors shaping couples’ participation in interventions aimed at improving maternal health outcomes [29].

McKeough et al. (2022) carried out a comprehensive database search to locate qualitative studies that explored the perceptions of pregnant or postpartum women regarding physical activity barriers and facilitators during pregnancy [30]. A total of 25 studies spanning 1985 to 2021 were reviewed, focusing on pregnant or postpartum women predominantly aged between 18 and 40, and the analysis identified 16 key themes. The most commonly mentioned barriers to physical activity included pregnancy-related symptoms, limited knowledge about safe activity practices, and the influence of opinions from the women’s social networks. On the other hand, frequently cited facilitators included social support and perceived benefits, encompassing physiological, psychological, and social advantages [30].

Sparks et al. (2023) examined the PA behaviors of pregnant women both quantitatively and quasi-qualitatively, aiming to better understand the obstacles and factors affecting adherence to physical activity guidelines during pregnancy [31]. A total of 431 women aged 18 and above participated in an online survey; the majority identified as White/Caucasian (84.5%), married (84.9%), and pregnant at the time of the study (66.6%). About 69.4% reported engaging in cardio-focused physical activity, and 77.4–88.8% expressed a willingness to follow physical activity recommendations for 2 to 5 days per week. The most commonly reported barriers included extreme fatigue (72.8%), physical discomfort (71.8%), and childcare responsibilities (57.8%). Conversely, key facilitators included the flexibility to choose the time of day (96.0%), access to home-based workout options (92.9%), and personalized exercise recommendations (95.6%) [31].

In 2024, May and colleagues examined the barriers and facilitators influencing physical activity among pregnant and postpartum Iranian women. Their review of 33 studies highlighted a range of challenges faced by these women in maintaining physical activity during this period which were grouped into three main categories: intrapersonal factors (including physical and emotional aspects), interpersonal factors (such as cultural constraints, social perceptions, and the influence of family members), and environmental factors (like organizational limitations and economic conditions). The review revealed that, although guidelines recommend physical activity for women without contraindications, less than 19% of women meet the suggested activity levels during pregnancy and postpartum, with the authors stressing the importance of healthcare professionals and social support from neighbors, family, and friends in promoting educational initiatives and reducing obstacles to physical activity [32].

In 2024, Dilworth et al. presented a comprehensive mixed-methods systematic review examining international clinical guidelines addressing smoking, nutrition, alcohol consumption, physical activity, and gestational weight gain (SNAP-W) within antenatal care across 14 countries [33]. The review encompassed 49 studies, comprising 27 qualitative and 22 quantitative investigations, and data from a cohort of 7146 antenatal care providers, including midwives, Aboriginal health workers, obstetricians, physicians, and general practitioners, leading to the identification of 352 barriers and enablers influencing clinical practice. Among the reviewed studies, the dominant domain within the Theoretical Domains Framework (TDF) was “environmental context and resources,” noted in 96% of studies, which highlights barriers such as time constraints, inadequate access to high-quality resources, and deficient organizational support. The second most frequently mentioned TDF domain was “beliefs about consequences,” reported in 67% of studies, with particular prominence in research concerning alcohol consumption, pregnancy-related nutrition, physical activity, and weight management, as well as in studies focused on midwives, multidisciplinary teams, and general practitioners. In contrast, “optimism” emerged as the second most prevalent domain in studies addressing tobacco-related care delivered by obstetricians, gynecologists, and other mixed health professionals [33].

Zhou et al. (2025) aimed to explore the extent of physical inactivity during early pregnancy and its associated factors among pregnant Chinese women [34]. Deploying the International Physical Activity Questionnaire, their findings revealed that 51.2% of participants experienced physical inactivity during early pregnancy, with walking being the most prevalent form of activity. Logistic regression analysis showed that women under 25 years of age were more likely to remain physically active compared to those aged 25–29 and 30–34. Additionally, women with higher educational attainment, such as a college or postgraduate degree, exhibited higher rates of inactivity compared to those with secondary education or less. Several factors contributing to increased physical inactivity were identified, including the absence of regular exercise prior to pregnancy, vaginal bleeding during early pregnancy, and poor sleep quality, with thematic analysis grouping these influencing factors into four categories: individual, interpersonal, social, and environmental. Common barriers included limited time, pregnancy-related discomfort, fear and anxiety, low self-confidence, insufficient resources, and contradictory advice. On the other hand, support from spouses and policies promoting or facilitating exercise during pregnancy emerged as key enablers of physical activity [34].

Efforts to quantify physical activity during pregnancy date back to 2006, when van de Pol et al. introduced the Pregnancy Physical Activity Questionnaire (PPAQ), specifically designed for pregnant women experiencing low back and hip pain. The PPAQ comprises 23 questions categorized into daily living (7 questions), household (10 questions), and outdoor activities (8 questions), with respondents indicating their level of difficulty performing these activities by selecting one of four options: 0 points for no difficulty, 1 for minor effort, 2 for significant effort, and 3 for requiring assistance. The Pregnancy Mobility Index (PMI) is calculated by summing the scores, providing healthcare professionals with a measure to monitor and evaluate physical activity during pregnancy [35]. Sattler et al. (2018) included 17 articles describing 18 studies of 11 different PA questionnaires (17 versions) in their systematic review [36]. Most versions of the questionnaires demonstrated insufficient measurement properties, particularly poor validity compared to accelerometers (Spearman’s ΔR ranging from 0.37 to 0.44) and reliance on self-report. They may also not accurately reflect low-intensity activity and a sedentary lifestyle. Only the French and Turkish versions of the Physical Activity in Pregnancy Questionnaire (PPAQ) demonstrated both sufficient reliability and construct validity. However, all combined versions of the PPAQ demonstrated insufficient construct validity. Despite these shortcomings, the authors recommend the PPAQ for assessing PA during pregnancy [36].

The objectification of barriers to physical activity is a separate issue. In 2021, Amiri-Farahani et al. developed and validated the Barriers to Physical Activity during Pregnancy Scale (BPAPS) [37]. After exploratory factor analysis led to the development of a 29-item scale, these items were divided into four main factors: pregnancy-related intrapersonal barriers, non-pregnancy-related intrapersonal barriers, interpersonal barriers, and environmental barriers. The scale demonstrated high internal consistency and stability—Cronbach’s alpha of 0.824 and a test–retest reliability of 0.87—indicating that this scale is a valid and appropriate tool for assessing barriers to physical activity in pregnant women [37]. Bor and Hanligil (2024) analyzed the reliability and validity of the Turkish version of the Barriers to Physical Activity in Pregnancy Scale (BPAPS) [38]. Quantitative research was conducted with 392 pregnant women aged 18 to 45 years. The Cronbach’s alpha coefficient for each subdimension was found to be greater than 0.70, and the overall scale had a Cronbach’s alpha coefficient of 0.91. The results indicated that the BPAPS used in the sample was a reliable measurement tool for the Turkish sample [38].

## 5. Discussion

Engaging in physical activity during pregnancy provides numerous benefits, including enhanced mental well-being and lower risks of gestational diabetes, preeclampsia, and excessive weight gain. Research strongly supports the safety of exercising during pregnancy, provided it is done at a moderate intensity and under the guidance of healthcare professionals.

Despite campaigns from organizations like the WHO and various national guidelines promoting physical activity in pregnant and postpartum women, many continue to underestimate its advantages. Studies reveal that most women tend to reduce their activity levels during this period, often influenced by specific barriers. Based on the socio-ecological model, these obstacles can be divided into three categories: intrapersonal (such as pregnancy-related symptoms, time limitations, lack of motivation, concerns about safety, and misconceptions about being active), interpersonal (including insufficient advice, limited information, and a lack of social support), and external factors like environmental, organizational, or political challenges (such as unfavorable weather conditions and access to resources). Addressing and progressively overcoming these issues requires coordinated efforts from healthcare providers and professionals.

Herazo-Beltrán et al. (2017) examined barriers to PA during pregnancy and postpartum, drawing comparisons to the general population, through a cross-sectional analysis involving 1066 adult women and 1036 adult men [39]. The study assessed various sociodemographic factors, including age, gender, marital status, socioeconomic status, and education level, alongside perceptions of barriers to engaging in PA, including insufficient time, limited social support, low energy levels, lack of motivation, inadequate skills, restricted resources, and concerns about exercise-related injuries. PA levels were measured using the abbreviated version of the International Physical Activity Questionnaire. The findings highlighted that lower-socioeconomic-status individuals faced heightened risks of perceiving barriers such as a lack of motivation and insufficient resources, while those with partners were less likely to perceive a lack of social support or motivation as obstacles to PA. Additionally, participants with lower educational attainment reported barriers including insufficient social support, limited resources, and fear of injury. Overall, factors such as socioeconomic status, marital status, educational background, and self-reported health were identified as significant predictors of perceived barriers to PA [39].

Garcia et al. (2022) conducted a comprehensive systematic review of studies investigating the relationship between physical activity (PA) levels and their modifiable barriers and facilitators, encompassing publications up to September 2020 [40]. The analysis identified 44 systematic reviews, with the majority of evidence concentrated on leisure-time PA, followed by travel-related PA, while limited review attention was given to PA within workplace, educational, and domestic settings. Across all PA domains, factors related to the built environment were examined more frequently than intra- or interpersonal factors. The review found consistently strong positive associations between various intra- and extra-personal factors and PA, and moderately consistent evidence pointed to positive correlations between PA and both family-based and general social support. Specific to transport-related PA, moderately consistent findings indicated positive associations with favorable beliefs about its outcomes, walkability, and the availability of infrastructure supporting active travel. The authors concluded that there remains a pressing need to broaden and deepen the scope of the evidence base concerning the barriers and facilitators influencing PA in less-explored domains, with the current predominant focus on leisure-related PA and built environment factors highlighting the necessity of more comprehensive approaches addressing domain-specific gaps [40].

Regarding healthcare, McKenna et al. (1998) shed light on barriers affecting PA promotion among general practitioners (GPs) and nurse practitioners (PNs) [41]. Through a survey of 419 GPs and 196 PNs identifying barrier types, impacts, stages of change in PA promotion, and respondents’ personal PA habits, the authors revealed that a significant proportion (69%) of GPs and PNs regularly encouraged PA among patients. However, GPs who cited insufficient time or lack of incentives were less inclined to promote PA, while those who were personally active were more likely to do so. A key insight from the study is that GPs in the “acting” or “maintaining” stage of change regarding their own physical activity are three times as likely to consistently promote similar behaviors to their patients compared to those in other stages; for PNs, this likelihood increases fourfold. Personal PA habits emerged as stronger predictors of readiness to promote PA than systemic barriers in both groups, and although systemic barriers noticeably influenced GPs’ readiness to implement such initiatives, this same effect was not observed in PNs [41].

When comparing the barriers to PA observed in the general population with those specific to the cohort of pregnant and postpartum women, it becomes evident that many obstacles are shared across both groups. However, certain barriers are uniquely associated with pregnancy and the postpartum period. Pregnancy barriers are largely dominated by physiological changes and fear for the baby’s safety, while postpartum barriers are defined by the demands of caring for the baby and lack of time for oneself. The main physical symptoms include fatigue, nausea, pain, dizziness, and discomfort, as well as difficulty moving due to weight gain and abdominal growth. Fears of miscarriage, premature birth, or harm to the baby are also present, often exacerbated by a lack of or inaccurate information. Sociocultural and structural barriers after childbirth include social perceptions (e.g., family discouraging activity), cultural beliefs, and a lack of specialized, safe exercise spaces. Limited advice from healthcare professionals regarding safe and appropriate levels of activity is also observed [32]. Important factors are also breastfeeding-related constraints, sleep disturbances, return to work, potential stress urinary incontinence, financial barriers, and levels of prior experience (distinguishing between primiparas and multiparas).

## 6. Study Limitations

This study has several limitations, including the reviewed authors’ use of varying methods to assess barriers to PA and differing views on the duration of the postpartum period (from six weeks to as long as a year). Confusion regarding the differentiation between physical activity, exercise, and recreational activity also contributes to methodological inconsistencies. Additionally, there are far too few studies assessing pregnancy- and postpartum-specific barriers to PA. To address these limitations, larger, longitudinal randomized controlled trials considering all possible barriers will be necessary.

## 7. Conclusions

A previous review found that physical activity (PA), as a non-pharmacological factor, is essential during pregnancy and the postpartum period, especially when undertaken at moderate to vigorous intensity. It is important to tailor PA programs to both women’s individual physical ability and the specific stage of pregnancy and the postpartum period.

The scientific literature reveals a significant lack of research on barriers to PA during pregnancy and the postpartum period, with challenges potentially stemming from personal issues, social influences, knowledge gaps, or environmental obstacles. Interestingly, these barriers tend to shift between pregnancy and the postpartum period, with safety concerns being more prevalent during pregnancy, while issues such as fatigue, lack of time, and childcare responsibilities dominate after delivery. This article uses a socio-ecological framework to analyze these barriers in depth, categorizing them within intra- and interpersonal, environmental, organizational, and policy levels. Further, more well-designed research is needed, through which other frameworks may be developed to explain these issues and the question of which forms of PA encounter the greatest barriers may be answered.

## Figures and Tables

**Table 1:** Absolute contraindications to exercise according to Canadian guideline [14].

1.	ruptured membranes,
2.	premature labour,
3.	unexplained persistent vaginal bleeding,
4.	placenta previa after 28 weeks gestation,
5.	preeclampsia,
6.	incompetent cervix,
7.	intrauterine growth restriction,
8.	high-order multiple pregnancy (e.g., triplets),
9.	uncontrolled Type I diabetes,
10.	uncontrolled hypertension,
11.	uncontrolled thyroid disease,
12.	other serious cardiovascular, respiratory or systemic disorder.

**Table 2:** Relative contraindications to exercise according to Canadian guideline [14].

1.	recurrent pregnancy loss,
2.	gestational hypertension,
3.	a history of spontaneous preterm birth,
4.	mild/moderate cardiovascular or respiratory disease,
5.	symptomatic anemia,
6.	malnutrition,
7.	eating disorder,
8.	twin pregnancy after the 28th week,
9.	other significant medical conditions.

**Table 3:** Main levels of socio-ecological model barriers impacting leisure-time physical activity among pregnant women [23,24,25,26,27,28,29,30,31,32,33,34].

Level	Descriptive Themes
Intrapersonal	Pregnancy-related symptoms and limitations
	Time constraints
	Perceptions of already being active
	Lack of motivation
	Mother–child safety concerns
Interpersonal	Lack of advice and information
	Lack of social support
Environmental, organizational, and policy	Adverse weather
	Lack of resources

## Data Availability

No new data were created in this study.
